# Supplementary material for: Democrats are better than Republicans at discerning true and false news but do not have better metacognitive awareness
Source: Commun Psychol. 2023 Dec 18;1:46. doi: 10.1038/s44271-023-00040-x (PMC11332161; doi:10.1038/s44271-023-00040-x)
Supplement: Supplementary file 1 — Supplementary Information [file 44271_2023_40_MOESM1_ESM.pdf]

Supplementary Materials

Supplementary Figure 1.

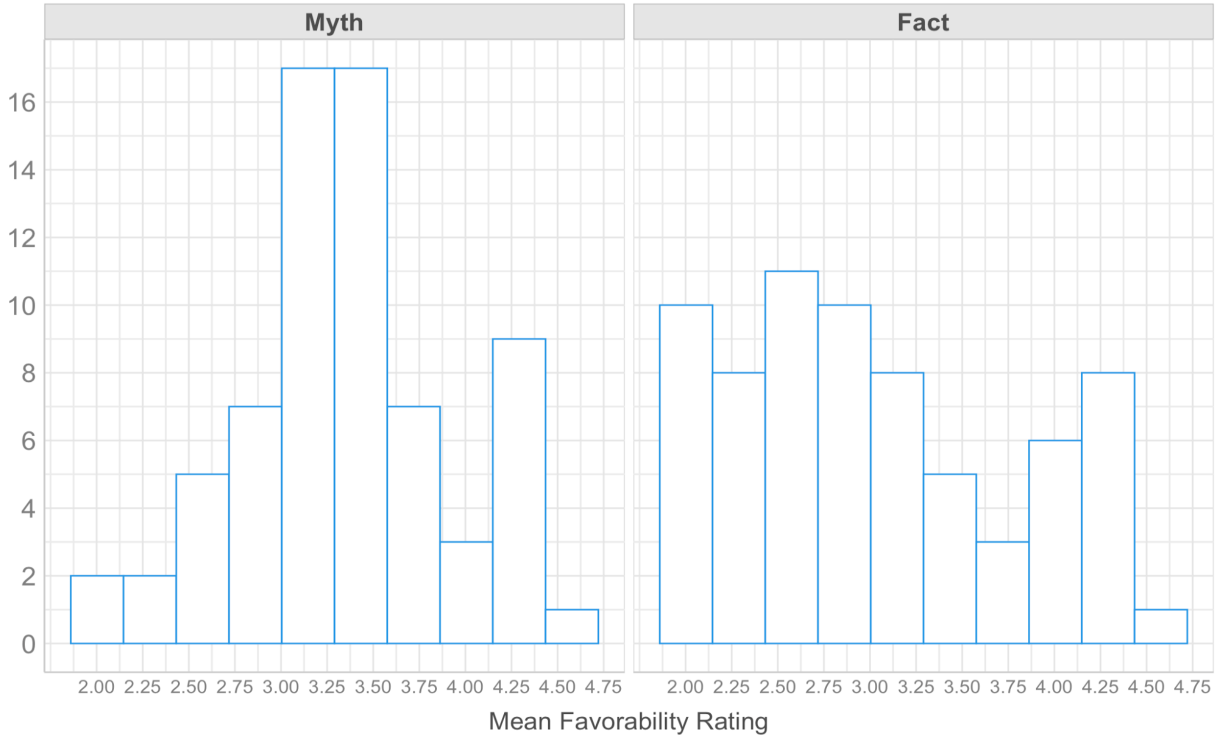

Supplementary Figure 1. Distribution of number of items by favorability rating from pilot study (*n* = 41 participants).

Supplementary Figure 2.

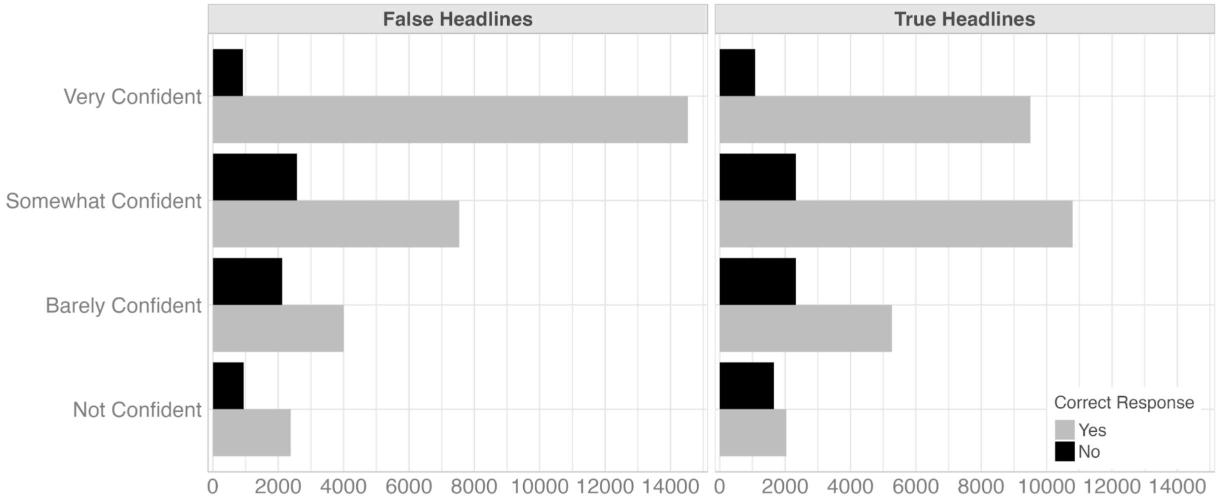

Supplementary Figure 2. Number of correct and incorrect responses, grouped by confidence level across the entire sample.

Supplementary Figure 3.

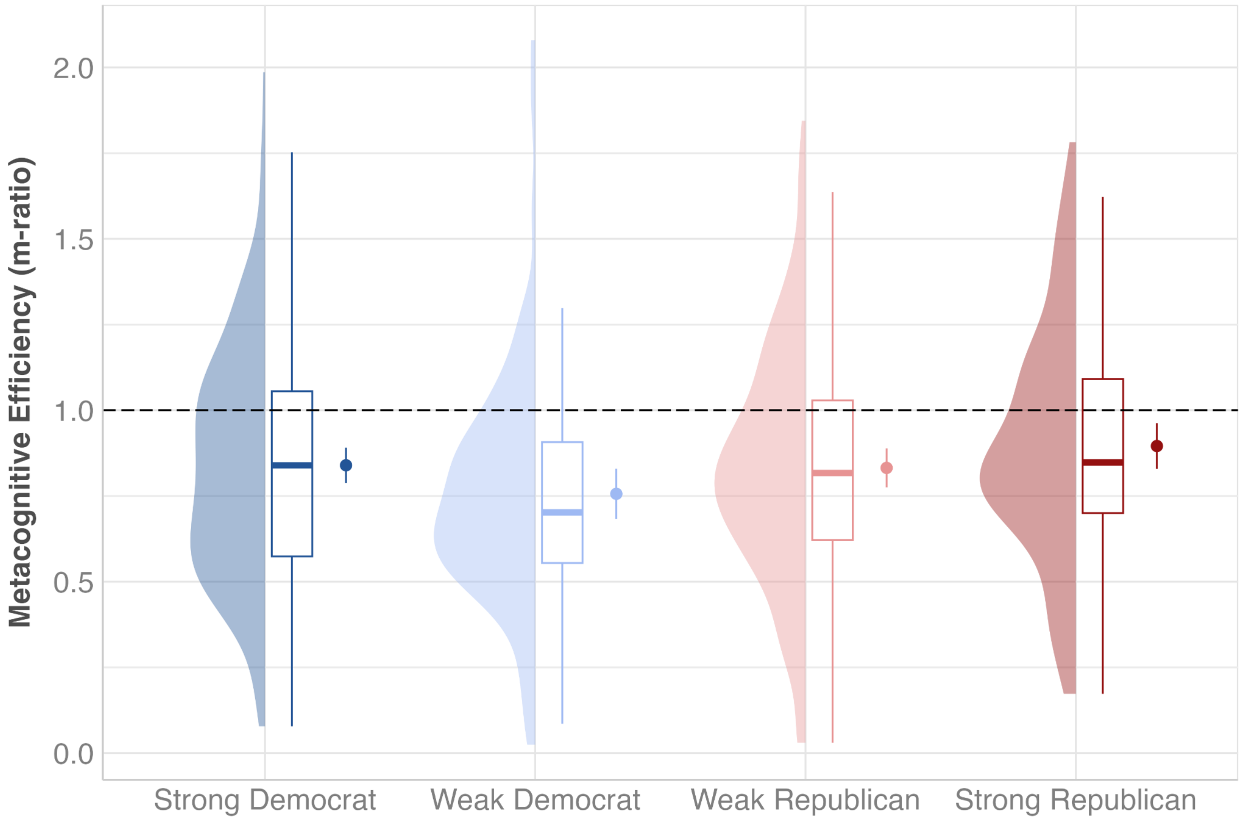

Supplementary Figure 3. Distributions, boxplots, and point estimates of metacognitive efficiency (i.e., *m-ratio* values) for each group using n = 64 items. Error bars represent 95% confidence intervals. Dotted line at y = 1 represents optimal metacognitive efficiency.

Supplementary Figure 4.

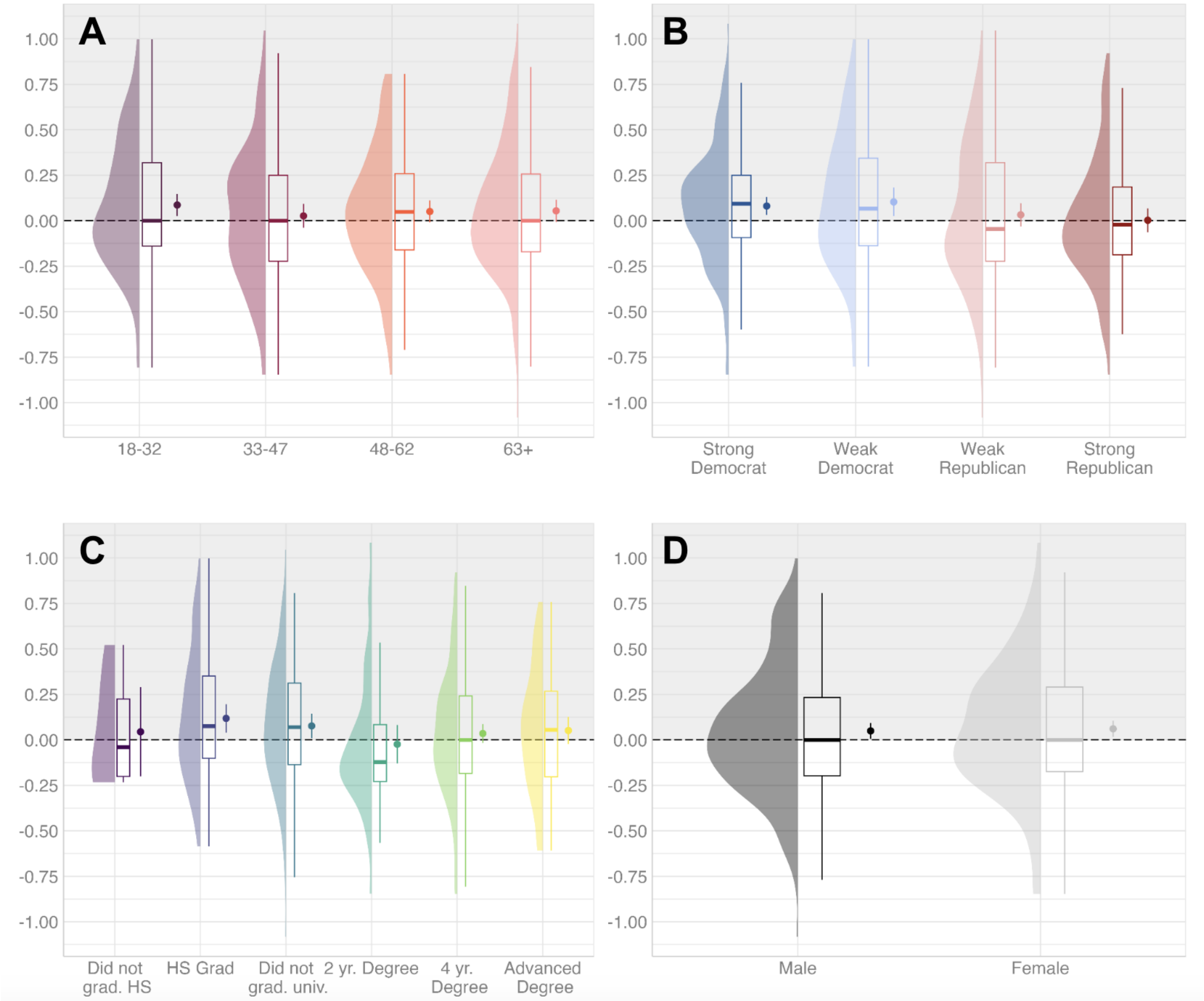

Supplementary Figure 4. Distributions, boxplots, and point estimates of response bias (i.e., *c* values) for each group. Error bars reflect 95% confidence intervals. Points in the gray area represent a tendency to answer false, and points in the white area represent a tendency to answer true. The dashed line at $c = 0$ represents no bias.

Supplementary Figure 5.

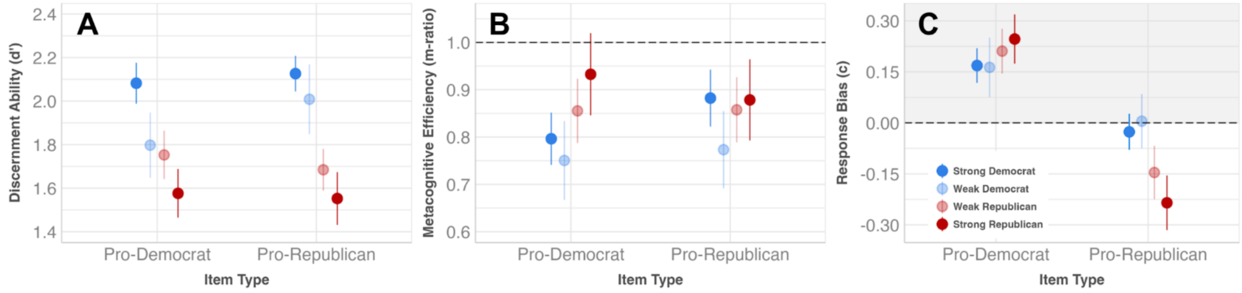

Supplementary Figure 5. Mean point estimates of discernment ability, metacognitive efficiency, and response bias for *n* = 64 equal items. Error bars reflect 95% confidence intervals. Points in the gray area represent a tendency to answer false, and points in the white area represent a tendency to answer true. The dashed line at *c* = 0 represents no bias. The dotted line at *y* = 1 in Panel B represents optimal metacognitive efficiency.

Supplementary Figure 6.

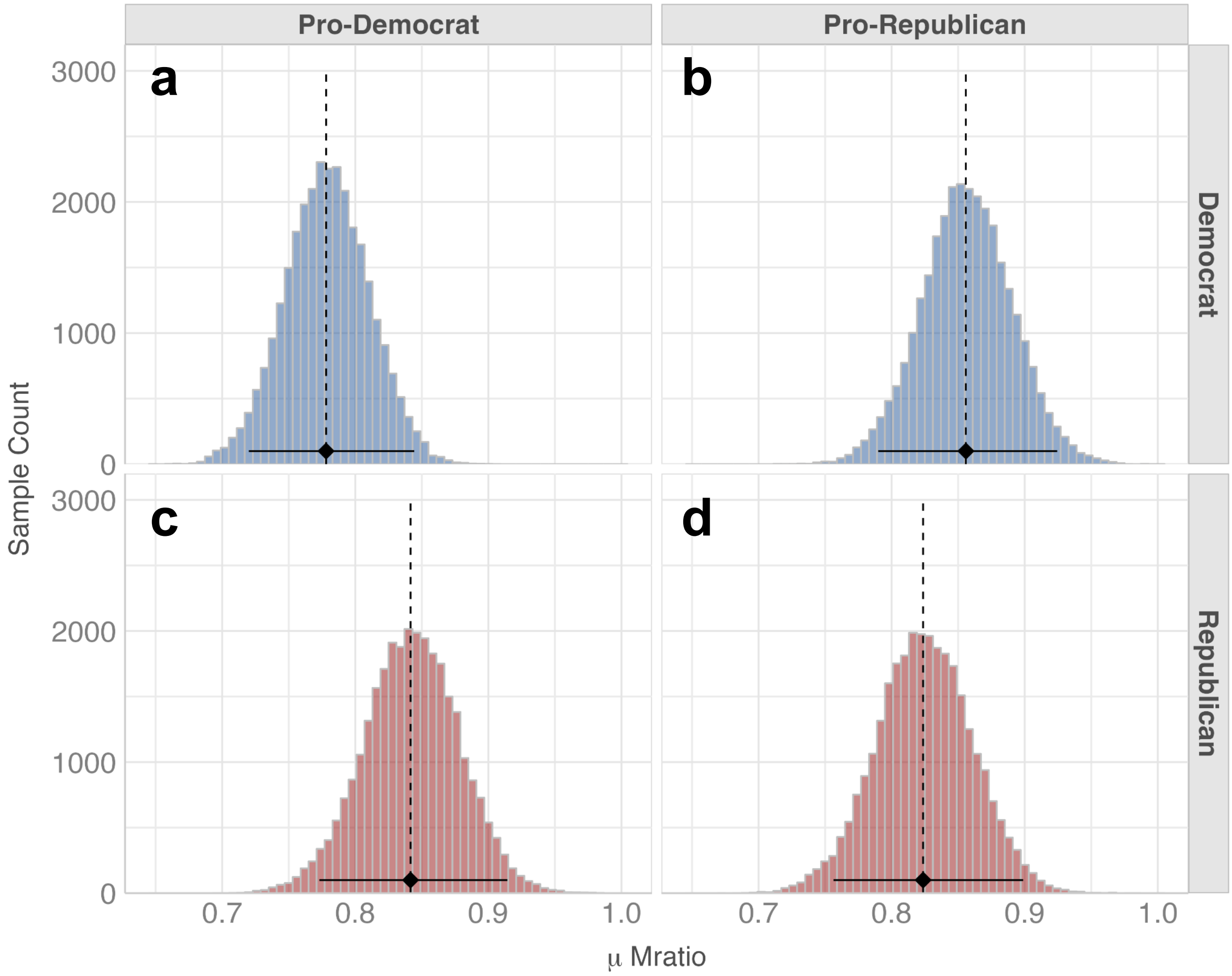

Supplementary Figure 6. Posterior distributions of group fit models for (a) Democrats on pro-Democrat items (*M* = .78, *95% HPDI* = [.720, .844]), (b) Republicans on pro-Democrat items (*M* = .84, *95% HPDI* = [.773, .914]), (c) Democrats on pro-Republican items (*M* = .86, *95% HPDI* = [.790, .924]), and (d) Republicans on pro-Republican items (*M* = .82, *95% HPDI* = [.756, .899]). Dashed vertical lines with point reflect means, and error bars reflect 95% HDPI's.

Supplementary Figure 7.

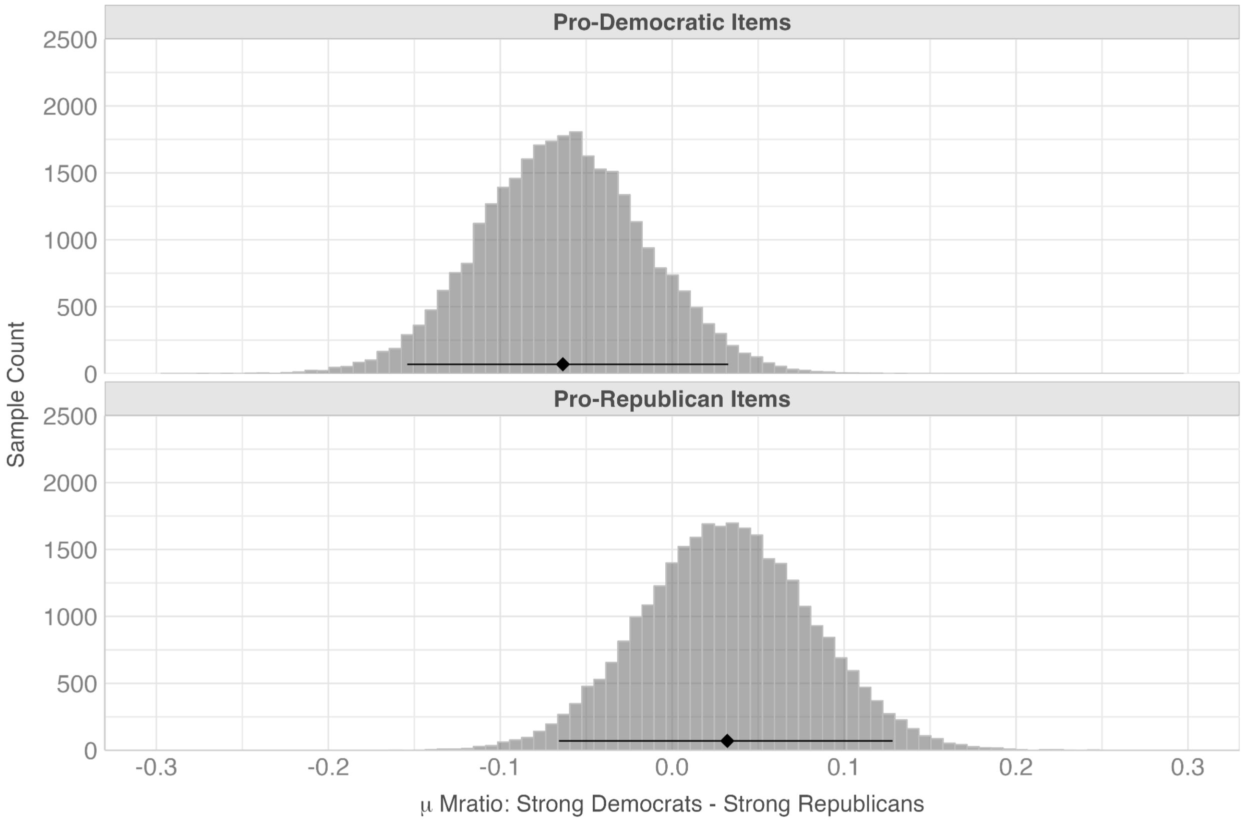

Supplementary Figure 7. Posterior distribution of the difference between strong Democrats' and strong Republicans' *m-ratio* values. Points reflect mean estimates, and error bars represent 95% highest density posterior intervals.

Supplementary Note 1

To assess differences between strong Democrats and strong Republicans in metacognitive efficiency estimated hierarchically at the group level, we subtract the posterior distribution of strong Republicans (*M* = .85, *95% HPDI* = [.804, .902]) from that of strong Democrats (*M* = .81, *95% HPDI* = [.758, .855]). As can be seen in Supplementary Figure 7, both 95% HDPI's cross 0, indicating that Democrats and Republicans do not differ from one another in metacognitive efficiency when these estimates are obtained using the hierarchical Bayesian approach to estimating *meta-d'*[1,2].

Supplementary Figure 8.

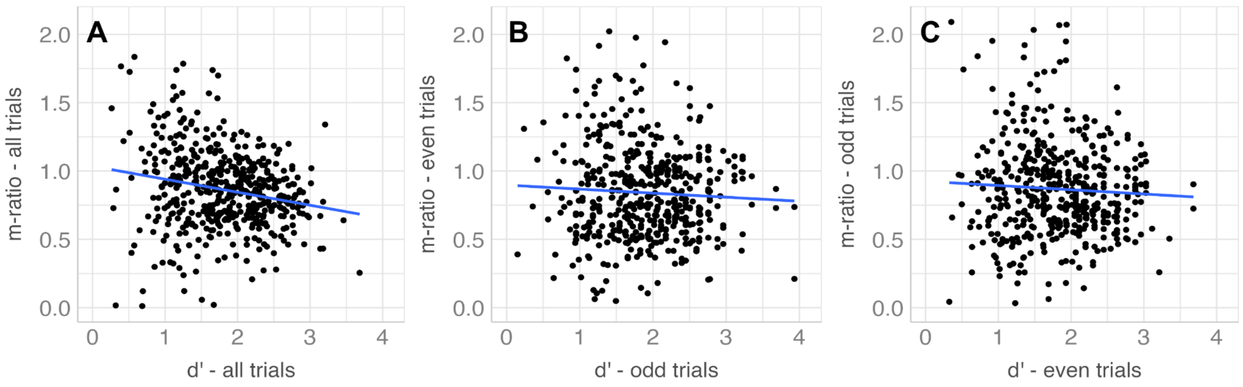

Supplementary Figure 8. Scatterplots of *d'* against *m-ratio* scores for all values (A), odd *d'* and even *m-ratio* values (B), and even *d'* and odd *m-ratio* values (C).

Supplementary Note 2

Because *d'* values are needed to calculate *m-ratio* values, the significant main effect of quartile reported in the main text may reflect a spurious correlation. For robustness, we used off *d'* trials to assess differences in even *m-ratio* trials, and vice versa. We split *d'* values into four quartiles, and eliminated outliers (n = 17) and participants with negative *m-ratio* values ($n = 10^{3-6}$). We then replicated the one-way ordinal ANOVA to compare the *m-ratio* values of each quartile on even and odd trials. For odd *d'* trials, we found no main effect of quartile (p = .227). Correlating participants' *d'* values on odd trials with *m-ratio* values on even trials replicated this result (p = .613). Repeating the one-way ordinal ANOVA to analyze differences in *m-ratio* values on odd trials produced a similar result (p = .461), as did correlating *d'* values on even trials with *m-ratio* values on odd trials (p = .485). Although this was inconsistent with the finding that the lowest scoring performers had the best metacognitive efficiency, it certainly does not support the finding that the least discerning participants have the *poorest* metacognitive efficiency.

Supplementary Methods

Pilot Study

We presented pilot participants (n = 41) with these claims and asked them to rate: 1) veracity (“Do you believe this claim to be true?” Yes/No), 2) confidence in their veracity judgment (“Are you confident in this choice?” Yes/No), and 3) the political leaning of the claim on a 5-point scale (“Assuming this claim is true, is it Pro-Democrat, Neutral, or Pro-Republican?”; 1 = Pro-Democrat; 3 = Neutral; 5 = Pro-Republican). Next, we removed 20 true and 20 false items nearing chance performance to ensure that the task was appropriately difficult to measure metacognitive ability (i.e., if the task were too difficult, variance in metacognition would be restricted by the entire sample performing poorly[7]). Finally, we attempted to better balance our stimuli while retaining as many items as possible by removing 10 pro-Republican and 10 pro-Democrat items, leaving 70 true (M = 3.02, SD = 1.24) and 70 false items (M = 3.36, SD = 1.25; see Figure S1).

Analysis Plan

Measuring discernment ability and response bias

To measure how effectively participants separated true from false information, as well as their general tendency to rate stimuli as true or false we utilized signal detection theory (SDT[8]). A basic SDT model requires participants to first provide a binary judgment about a given stimuli (e.g., true vs. false for a given headline) and then rate their confidence in this judgment. Using these type-1 judgments – where type 1 refers to accuracy on the binary choice task – type 1 hit and false alarms rate can be calculated (see Table 1). These rates can then be used to calculate a discernment ability measure (“d prime” or *d’*), and a response bias measure (*c* and/or *c’*). The term discernment ability (or discrimination sensitivity more generally) refers to how effectively participants distinguish between two classes of stimuli, whereas the term response bias measures their tendency to provide the same binary judgment (in this case true or false) across all trials. The specific formulas for calculating each of these indices can be found in our preregistration. Due to a transcription error in our preregistration, the updated formula for calculating *c* values is presented below, where *z* represents a given hit or false alarm rate’s value in an inverse cumulative distribution function:

$$c = -.5 \times [z(Hit\ Rate) + z(False\ Alarm\ Rate)]$$

An important advantage of SDT is its ability to treat discernment ability and response bias as distinct processes. For example, when assessing how accurately a participant can discern true from false news headlines, it is useful to look at their number of hits, or the number of true trials that they classify as such. However, by looking only at this category, one cannot be sure if the participant is actually effective at discriminating between the two categories, or simply maintains a general tendency to rate all stimuli as true. SDT’s solution is the *d’* index for

measuring discernment ability, and *c* for measuring the propensity to provide a particular response for all stimuli.

Defining metacognitive sensitivity and efficiency

To measure metacognitive sensitivity, we use Maniscalco and Lau's *meta-d'* approach[9,10]. Also based in SDT, the meta-d' model uses participants' confidence ratings about their binary judgments to estimate *type-2* hit and false alarm rates, where type 2 refers to the accuracy of expressed confidence ratings instead of accuracy on the task itself (i.e., hits expressed with high confidence, false alarms expressed with low confidence, etc. See Table 1). From here, a type 2 parallel to *d'* can be computed, called *meta-d'*. *Meta-d*' can be interpreted as the *d'* value (or type 1 performance level) that a participant would need to produce to be considered metacognitively ideal (i.e., expressing high confidence in every hit, low confidence in every false alarm, etc.) based on their confidence ratings.

Prior work demonstrates that common measures of metacognitive sensitivity (e.g., phi/Gamma correlations; AUROC values) are bound by both type-1 performance and type-1 response bias artifacts[1, 11]. In other words, overall task performance is often correlated with awareness of performance (e.g., correctly responding to a stimulus is often positively related to how aware one is that they responded correctly). However, the meta-d' framework is capable of sidestepping both of these. Unlike confidence-accuracy correlations, *meta-d'* can separate how well a participant makes metacognitive judgments (i.e., their metacognitive sensitivity) from their general tendency to respond with high or low confidence across all trials (i.e., their metacognitive bias[12]) by using separate indices. Additionally, because *meta-d'* is measured in *d'* units, these two terms can be easily compared. Simply dividing *meta-d'* by *d'* removes the type-1 performance confound, producing a measure of metacognitive efficiency called the *m-ratio*. This value can be thought of as a participant's metacognitive sensitivity given a specific level of task performance – in other words, how metacognitively capable (or efficient) one is given the difficulty of the task. A metacognitively ideal observer (i.e., a participant answering trials correctly with high confidence and incorrectly with low confidence) would thus have a *meta-d'* value equivalent to their *d'* value, producing an *m-ratio* of 1.

Supplementary Results

Meta-d' Results

Age

To assess differences in metacognitive sensitivity across partisanship, age, education, and gender, one-way ordinal ANOVA with the factor age group on *meta-d'* values. We found no significant differences in *meta-d'* values across age groups (p = .117). This suggests that participants in each age bin were similarly aware of their ability to discern true and false headlines.

Partisanship

After conducting a 2 × 2 factorial ANOVA with between-subjects factors partisanship and partisanship strength on meta-*d'* values, we found a significant main effect of partisanship (F (1, 494) = 74.44, p < .001, MSE = .31, ηp^2 = .13), partisanship strength (F (1, 494) = 4.90; p = .027, MSE = .31, ηp^2 = .01), and their interaction (F (1, 494) = 29.99, p < .001, MSE = .31, ηp^2 = .06).

Education and Gender

Finally, we examined differences in *meta-d'* values according to education and gender. We correlated education and *meta-d'* values and found a positive relationship between the two (ρ = .25, p < .001). An independent samples t-test revealed that men in our sample were more metacognitively sensitive than women (t(497) = 2.66, p = .008, 95% CI = [.038, .257], Cohen's d = .24).

c' Results

The formulas that we reported for calculating this value in our preregistration were incorrect due to a transcription error. *c*' values represent participants' response bias criterion adjusted for their actual performance.

Age

Repeating the one-way ordinal ANOVA with age group as a factor (18-32, 33-47, 48-62, 63+) on *c'* values revealed no evidence of differences in response bias across age groups, (p = .267). In other words, participants' propensity to evaluate a headline as true or false does not appear to depend on age, controlling for their performance.

Partisanship

Turning our investigation to partisanship, we repeated the 2 × 2 factorial ANOVA with between-subjects factors partisanship (Democrat vs. Republican) and partisanship strength (strong partisan vs. weak partisan) described above on *c'* values. Although we found a main effect of partisanship (F (1, 485) = 5.25; p = .022; MSE = .05; ηp^2 = .01), we did not find a main effect of partisanship strength (p = .356) or a partisanship × partisanship strength interaction (p = .783).

Education and Gender

Finally, we examined differences in *c*' values according to education and gender. We correlated education and *c*' values and found no relationship between the two ($\rho = -.05$, $p = .236$). An independent samples t-test revealed that men and women did not differ in their response bias adjusted for performance ($p = .877$).

Misinformation Susceptibility Test

Finally, we assessed the predictive validity of the Misinformation Susceptibility Test[13]. We calculated a veracity discernment measure for each participant by summing their total number of correct responses on the MIST-20. We then used this sum to predict participants' *d'* value on non-MIST trials ($n = 125$). We found that veracity discernment as calculated by the MIST was a strong predictor of discerning true from false headlines on our task ($\beta = .18$, $SE = .01$, $t(498) = 20.5$, $p < .001$; $R^2 = .41$). In other words, people's performance on 20 MIST items predicted 41% of the variance in their discernment ability of the remaining 125 items.

Supplementary Table 1. Items included in stimuli set(s) with favorability ratings

Item	Claim	Veracity	Avg Favorability	Party Favorability	Included in Equated
1	Americans are easing up on masks and other COVID safeguards according to AP Poll	TRUE	3.39	Republican	Yes
2	Sarah Palin seeks return to national politics by launching bid for House seat	TRUE	3.68	Republican	Yes
3	International relations experts and U.S. public agree: America is respected less globally	TRUE	3.07	Republican	Yes
4	Russia said that the U.S. is "adding fuel to the fire" by providing weapons for Ukraine	TRUE	3.39	Republican	Yes
5	Several airlines are pushing the White House to end COVID testing requirements	TRUE	3.56	Republican	Yes
6	An irreplaceable $2 million dollar tabernacle was stolen from a Catholic church in Brooklyn	TRUE	3.05	Republican	Yes
7	Los Angeles to ban homeless camps near schools as crisis spreads	TRUE	3.1	Republican	Yes
8	Gas prices are higher than $8 per gallon at some California gas stations	TRUE	3.46	Republican	Yes
9	Iran's enriched uranium now 18 times more than the nuclear deal's limit, U.N. watchdog says	TRUE	3.17	Republican	Yes
10	Democratic congressman Ro Khanna says Biden can do "way more" to fight inflation	TRUE	3.2	Republican	Yes
11	Facebook censors senator after she posted that "biological men have no place in women's sports"	TRUE	3.22	Republican	Yes
12	North Carolina lawmakers advance a bill limiting LGBTQ teachings in schools	TRUE	4.46	Republican	Yes
13	Elon Musk tells Tesla workers, "return in person or resign"	TRUE	3.17	Republican	Yes
14	Republican Congresswoman Lauren Boebert on gun control:	TRUE	3.68	Republican	Yes

	"When 9/11 happened, we didn't ban planes"				
15	U.S. inflation hit 8.6% in May 2022	TRUE	3.34	Republican	Yes
16	Women's basketball star Brittney Griner seen in court, Russia extends detention	TRUE	3.05	Republican	Yes
17	Biden ordered an 'unprecedented' release of oil reserves	TRUE	2.56	Democrat	Yes
18	Europe and the U.S. are making ambitious plans to reduce reliance on Russian gas	TRUE	2.73	Democrat	Yes
19	Florida man sentenced to 18 months in prison for Pelosi, AOC death threats	TRUE	2.56	Democrat	Yes
20	Pence slams Trump for 'un-American' bid to overturn vote	TRUE	2.71	Democrat	Yes
21	Pentagon links leadership failures to violence, harassment, at military bases	TRUE	2.76	Democrat	Yes
22	Attitudes towards EU are largely positive, both within Europe and outside of it	TRUE	2.78	Democrat	Yes
23	Hyatt will remove small bottles from hotel bathrooms by 2021	TRUE	2.73	Democrat	Yes
24	Republicans divided in views of Trump's conduct, Democrats broadly critical	TRUE	2.66	Democrat	Yes
25	Guns banned during Trump's speech at the NRA conference in Texas post-Uvalde	TRUE	2.56	Democrat	Yes
26	COVID vaccines finally available for children under 5	TRUE	2.49	Democrat	Yes
27	The job market stays red-hot with unemployment rates near pre-pandemic low	TRUE	2.46	Democrat	Yes
28	Apple store workers vote to form the company's first U.S. union	TRUE	2.46	Democrat	Yes
29	Biden commits U.S. support to Finland and Sweden joining NATO	TRUE	2.51	Democrat	Yes
30	Education Department to cancel $5.8 billion in debt for students of Corinthian Colleges	TRUE	2.46	Democrat	Yes
31	Admiral Linda Fagan becomes the first woman to lead a U.S. armed forces branch	TRUE	2.68	Democrat	Yes
32	Democratic states are extending welfare benefits to	TRUE	2.83	Democrat	Yes

	undocumented individuals				
33	21,000 dead people registered on Pennsylvania's voter rolls	FALSE	4.29	Republican	Yes
34	Governors' conference votes 32-18 to impeach Nancy Pelosi	FALSE	4.32	Republican	Yes
35	A new study shows a clear relationship between eye color and intelligence	FALSE	3.12	Republican	Yes
36	According to a new study, the Ebola virus was caused by U.S. nuclear weapons testing	FALSE	3.1	Republican	Yes
37	Illinois Democrats pass bill forcing kids to attend LGBT classes	FALSE	3.12	Republican	Yes
38	Nancy Pelosi stops a bill to make English the official language of the U.S.	FALSE	3.24	Republican	Yes
39	Rutgers University declares English grammar racist	FALSE	3.24	Republican	Yes
40	Gun manufacturing is the only industry in the U.S. exempt from being sued	FALSE	3.15	Republican	Yes
41	Justin Trudeau tells Canadians to stop talking to their unvaccinated relatives	FALSE	3.2	Republican	Yes
42	Merriam-Webster changes its dictionary definition of "racism" to show that only white people are racist	FALSE	3.22	Republican	Yes
43	Supreme Court Justice Ketanji Brown Jackson: "judges should use critical race theory in making their rulings"	FALSE	3.12	Republican	Yes
44	Biden is wanted in Ukraine on Class A felony charges	FALSE	4.44	Republican	Yes
45	A Tennessee bill would make it easier for adults to marry children if passed	FALSE	3.12	Republican	Yes
46	CDC revealed to be private non-profit organization	FALSE	3.22	Republican	Yes
47	Products from Bath and Body Works are suspected of damaging fertility	FALSE	3.15	Republican	Yes
48	San Francisco to rename Abraham Lincoln High School because Lincoln 'didn't show that black lives matter'	FALSE	3.2	Republican	Yes
49	Dick Cheney hanged at Guantanamo Bay	FALSE	2.95	Democrat	Yes
50	Fauci awarded Nobel Peace Prize	FALSE	2.15	Democrat	Yes
51	Giuliani prepares for new role as MyPillow pitchman	FALSE	2.93	Democrat	Yes

52	Portland bans urinals in public buildings out of respect to the city's "shared values"	FALSE	2.76	Democrat	Yes
53	Georgia Republicans move Atlanta voting booth to icy mountain top	FALSE	2.59	Democrat	Yes
54	Republicans to support Democratic leader being chosen based on race instead of merit	FALSE	2.83	Democrat	Yes
55	Rupaul claims Trump touched him inappropriately during the 1990's	FALSE	2.07	Democrat	Yes
56	Donald Trump claims he hardly knows Donald Trump Jr.	FALSE	2.49	Democrat	Yes
57	Trump threatens to sue founding fathers	FALSE	2.32	Democrat	Yes
58	The Ford Foundation donates millions to the "defund the police" movement	FALSE	2.49	Democrat	Yes
59	Fox News host Jesse Watters calls for the assassination of Dr. Fauci	FALSE	2.95	Democrat	Yes
60	Biden's climate plan includes cutting 90% of red meat from Americans' diets by 2030	FALSE	2.98	Democrat	Yes
61	Biden: "white Republican men are more dangerous than the Islamic State group"	FALSE	2.68	Democrat	Yes
62	Joe Biden approves new card that gives free health insurance to Americans who are 25 and older	FALSE	1.93	Democrat	Yes
63	U.S. Supreme Court Justice Amy Coney Barrett placed under house arrest by military investigators	FALSE	2.68	Democrat	Yes
64	Elon Musk to donate $250 billion for reparations to African Americans in U.S.	FALSE	2.63	Democrat	Yes
65	21 states sued the Biden Administration to end the federal travel mask mandate	TRUE	4.2	Republican	No
66	Arizona Republicans recently enacted a controversial new proof-of-citizenship voting law	TRUE	4.05	Republican	No
67	Biden's approval ratings have recently plummeted amid war and inflation fears according to a new public opinion poll	TRUE	4.07	Republican	No
68	Inflation gets a starring role in GOP Congressional campaign	TRUE	4.24	Republican	No

	ads				
69	Oklahoma, Arizona governors sign transgender sports ban	TRUE	4.39	Republican	No
70	Supreme Court sides with Republicans in case on Wisconsin redistricting	TRUE	4.37	Republican	No
71	Utah bans transgender athletes in girls' sports despite governor's veto	TRUE	*4.05*	Republican	No
72	Supreme Court overturns Roe v. Wade	TRUE	*4.29*	Republican	No
73	New GOP platform in Texas calls for the U.S. to leave the United Nations	TRUE	4.00	Republican	No
74	Nancy Pelosi's husband arrested for drunk driving	TRUE	3.95	Republican	No
75	Trump says, "U.S. should fund safe schools before Ukraine"	TRUE	3.95	Republican	No
76	Trump says, "Mass shootings are a reason to arm people, not disarm them"	TRUE	4.29	Republican	No
77	Supreme Court ruling expands U.S. gun rights	TRUE	4.22	Republican	No
78	Supreme Court supports football coach's right to pray on the 50 yard-line before games	TRUE	4.2	Republican	No
79	Sarah Palin currently leading in a special election for House seat in Alaska	TRUE	3.78	Republican	No
80	Senate panel approves Jerome Powell's renomination as chairman of the Federal Reserve	TRUE	2.98	Democrat	No
81	Rep. Alexandria Ocasio-Cortez calls for Justice Clarence Thomas to resign	TRUE	2.07	Democrat	No
82	Stewart Rhodes added to Jan. 6 lawsuit against Proud Boys, Oath Keepers	TRUE	2.27	Democrat	No
83	Trump swooped in to profit from White House photographer's book deal	TRUE	2.15	Democrat	No
84	Biden on jobs report: "Americans are back to work"	TRUE	1.9	Democrat	No
85	One-in-three worldwide lack confidence in NGO's (non-governmental organizations)	TRUE	3.00	Democrat	No
86	Reflecting a demographic shift, 109 U.S. counties have become	TRUE	2.37	Democrat	No

	majority non-white since 2000				
87	Global warming age gap: Younger Americans most worried	TRUE	1.95	Democrat	No
88	U.S. support for legal marijuana steady in the past year	TRUE	2.29	Democrat	No
89	In Britain, it took just one school shooting to pass major gun control legislation	TRUE	1.98	Democrat	No
90	Ted Cruz walks away from reported who asked why the U.S. has so many mass shootings	TRUE	2.27	Democrat	No
91	Twitter fined $150 million in the U.S. for selling users' data	TRUE	3.00	Democrat	No
92	Biden considering temporarily suspending federal gas tax of 18.4 cents per gallon	TRUE	1.95	Democrat	No
93	Biden announces new rockets and munitions to Ukraine	TRUE	2.39	Democrat	No
94	Massachusetts agrees to a legal settlement for people who were wrongfully convicted of drug charges	TRUE	2.32	Democrat	No
95	Strawberries recalled in connection with Hepatitis A outbreak	TRUE	3.00	Democrat	No
96	Kim Kardashian called for stricter gun laws after Texas shooting	TRUE	2.05	Democrat	No
97	Laverne Cox is the first transgender woman to have a Barbie doll modeled after her	TRUE	2.00	Democrat	No
98	Michigan candidate for governor Ryan Kelley charged for Jan. 6 involvement	TRUE	2.44	Democrat	No
99	Leader of the Proud Boys charged with seditious conspiracy	TRUE	2.07	Democrat	No
100	New York announces they will let students use chosen names and pronouns for college records and diplomas	TRUE	2.1	Democrat	No
101	Meta/Facebook rolls out additional parental controls for Instagram and virtual reality	TRUE	2.9	Democrat	No
102	Recent survey shows that majority of Texas teachers do not want to be armed in classroom	TRUE	2.05	Democrat	No
103	Ballots in Arizona's Maricopa County found shredded and in dumpster days before Senate audit	FALSE	3.73	Republican	No
104	Whistleblower drops hard evidence that Biden, Obama, and	FALSE	4.29	Republican	No

	Hillary executed Seal Team 6				
105	Bill Clinton brought his teenage daughter to Epstein's private island	FALSE	3.76	Republican	No
106	U.N. health experts admit toxic vaccine ingredients are harming children worldwide	FALSE	3.83	Republican	No
107	Malia Obama arrested again, complete media blackout	FALSE	4.22	Republican	No
108	Military: "Biden has NO nuclear launch codes"	FALSE	3.51	Republican	No
109	Military defies Biden regime, refuses to deploy	FALSE	3.83	Republican	No
110	Military: Fauci likes young boys	FALSE	4.2	Republican	No
111	Michelle Obama files for divorce after shocking revelation; Barack Obama admits, "I'm gay!"	FALSE	4.15	Republican	No
112	The corporate media is controlled by the military-industrial complex: The major oil companies own the media and control their agenda	FALSE	3.39	Republican	No
113	A new study shows that left-wingers are more likely to lie to get a higher salary	FALSE	3.93	Republican	No
114	The government is manipulating the public's perception of genetic engineering in order to make people more accepting of such techniques	FALSE	3.66	Republican	No
115	According to a UN report, left-wing extremism causes more damage to the world than terrorism	FALSE	4.07	Republican	No
116	Government officials have illegally manipulated the weather to cause devastating storms	FALSE	3.54	Republican	No
117	Ukraine President Zelenskyy's cousin is billionaire investor George Soros	FALSE	3.61	Republican	No
118	Wisconsin school district sets "furry protocols" that allow students to identify as cats	FALSE	3.44	Republican	No
119	Queen Elizabeth takes Ivermectin to treat COVID-19	FALSE	3.83	Republican	No
120	Astronaut Buzz Aldrin admits during a television appearance that the moon landing was fake	FALSE	3.27	Republican	No
121	The Michigan House of Representatives passed human	FALSE	3.41	Republican	No

	microchipping legislation				
122	Nancy Pelosi's son arrested for murder	FALSE	3.98	Republican	No
123	Nancy Pelosi taken from her office in handcuffs	FALSE	4.29	Republican	No
124	Republican senator unveils plan to send all U.S. teachers to Marine bootcamp	FALSE	3.34	Republican	No
125	McDonald's to charge Caucasian customers a $1.50 service fee	FALSE	3.41	Republican	No
126	During Trump's presidency, he completely refilled the U.S. emergency stockpile of petroleum	FALSE	4.29	Republican	No
127	New York hospitals are not releasing babies to unvaccinated parents	FALSE	3.44	Republican	No
128	A university in Canada has banned the use of capital letters to avoid scaring students	FALSE	3.27	Republican	No
129	The U.S. government is paying farmers to destroy crops to create a food shortage	FALSE	3.34	Republican	No
130	Counties in the U.S. can access CDC and Census data to identify unvaccinated individuals	FALSE	3.29	Republican	No
131	The World Health Organization proposes ban on women of childbearing age drinking alcohol	FALSE	3.37	Republican	No
132	NPR began reporting on the January 6th storming of the capitol before it started	FALSE	3.68	Republican	No
133	Meta/Facebook CEO Zuckerberg under investigation for bribing government officials to influence 2020 election	FALSE	3.39	Republican	No
134	Ukrainian soldiers seen propping up "wounded" dummy for war propaganda video	FALSE	3.41	Republican	No
135	Medicare to go bankrupt in 4 years	FALSE	3.56	Republican	No
136	Untapped oil reserves un the U.S. exceed those in Saudi Arabia	FALSE	3.44	Republican	No
137	Swedish climate activist Greta Thunberg urges Chinese people to stop using chopsticks in order to save trees	FALSE	3.27	Republican	No
138	Disney CEO Bob Chapek arrested for human trafficking	FALSE	3.39	Republican	No
139	The news agency Reuters revealed to be a Russian-owned	FALSE	3.37	Republican	No

	company				
140	Not a single person in the crowd on January 6th was found to be carrying a firearm	FALSE	4.22	Republican	No
141	Morocco's king appoints committee chief to fight poverty and inequality	TRUE	*NA*	*NA*	No
142	Democrats more supportive than Republicans of federal spending for scientific research	TRUE	*NA*	*NA*	No
143	Government officials have manipulated stock prices to hide scandals	FALSE	*NA*	*NA*	No
144	Certain vaccines are loaded with dangerous chemicals and toxins	FALSE	*NA*	*NA*	No
145	The government is knowingly spreading disease through the airwaves and food supply	FALSE	*NA*	*NA*	No

Note: NA values are listed for Misinformation Susceptibility Test items not included in the pilot study[13]).

Supplementary References

1. Fleming, S. M. HMeta-d: Hierarchical Bayesian estimation of metacognitive efficiency from confidence ratings. Neuroscience of Consciousness, (2017).
2. Mazancieux, A., Fleming, S. M., Souchay, C., & Moulin, C. J. Is there a G factor for metacognition? Correlations in retrospective metacognitive sensitivity across tasks. Journal of Experimental Psychology: General, 149, 1788-1799, (2020).
3. Burson, K. A., Larrick, R. P., & Klayman, J. Skilled or unskilled, but still unaware of it: How perceptions of difficulty drive miscalibration in relative comparisons. Journal of Personality and Social Psychology, 90, 60–77 (2006).
4. Feld, J., Sauermann, J., & De Grip, A. Estimating the relationship between skill and overconfidence. Journal of Behavioral and Experimental Economics, 68, 18-24 (2017).
5. Hoaglin, D. C., & Iglewicz, B. Fine-tuning some resistant rules for outlier labeling. Journal of the American Statistical Association, 82, 1147-1149, (1987).
6. Guggenmos, M. Measuring metacognitive performance: type 1 performance dependence and test-retest reliability. Neuroscience of consciousness, 2021(1), niab040 (2021).
7. McIntosh, R. D. et al. Wise up: Clarifying the role of metacognition in the Dunning-Kruger effect. Journal of Experimental Psychology: General, 148, 1882–1897, (2019).
8. Hautus, M. J., Macmillan, N. A., & Creelman, C. D. Detection theory: A user's guide. (Routledge, 2021).
9. Maniscalco, B., & Lau, H. A signal detection theoretic approach for estimating metacognitive sensitivity from confidence ratings. Consciousness and Cognition, 21(1), 422–430 (2012).

10. Maniscalco, B., & Lau, H. The Cognitive Neuroscience of Metacognition. Signal Detection Theory Analysis of Type 1 and Type 2 Data: Meta-*d′*, Response-Specific Meta-*d′*, and the Unequal Variance SDT Model. (eds. Fleming, S., & Frith, C.) (Springer, 2014).

11. Galvin, S. J., Podd, J. V., Drga, V., & Whitmore, J. Type 2 tasks in the theory of signal detectability: Discrimination between correct and incorrect decisions. Psychonomic Bulletin & Review, 10, 843–876 (2003).

12. Fleming, S. M., & Lau, H. C. How to measure metacognition. Frontiers in human neuroscience, 8, (2014).

13. Maertens, R., et al. The Misinformation Susceptibility Test (MIST): A psychometrically validated measure of news veracity discernment. Behavior Research Methods (2023).
