# Supplementary material for: Democrats are better than Republicans at discerning true and false news but do not have better metacognitive awareness
Source: Commun Psychol. 2023 Dec 18;1:46. doi: 10.1038/s44271-023-00040-x (PMC11332161; doi:10.1038/s44271-023-00040-x)
Supplement: Supplementary file 3 — Reporting Summary [file 44271_2023_40_MOESM3_ESM.pdf]

nature portfolio

Corresponding author(s): Briony Swire-Thompson
Last updated by author(s): Nov 1, 2023

Reporting Summary

Nature Portfolio wishes to improve the reproducibility of the work that we publish. This form provides structure for consistency and transparency in reporting. For further information on Nature Portfolio policies, see our Editorial Policies and the Editorial Policy Checklist.

Statistics

For all statistical analyses, confirm that the following items are present in the figure legend, table legend, main text, or Methods section.

n/a	Confirmed	
☐	☒	The exact sample size (*n*) for each experimental group/condition, given as a discrete number and unit of measurement
☐	☒	A statement on whether measurements were taken from distinct samples or whether the same sample was measured repeatedly
☐	☒	The statistical test(s) used AND whether they are one- or two-sided<br>*Only common tests should be described solely by name; describe more complex techniques in the Methods section.*
☒	☐	A description of all covariates tested
☐	☒	A description of any assumptions or corrections, such as tests of normality and adjustment for multiple comparisons
☐	☒	A full description of the statistical parameters including central tendency (e.g. means) or other basic estimates (e.g. regression coefficient) AND variation (e.g. standard deviation) or associated estimates of uncertainty (e.g. confidence intervals)
☐	☒	For null hypothesis testing, the test statistic (e.g. *F*, *t*, *r*) with confidence intervals, effect sizes, degrees of freedom and *P* value noted<br>*Give P values as exact values whenever suitable.*
☐	☒	For Bayesian analysis, information on the choice of priors and Markov chain Monte Carlo settings
☐	☒	For hierarchical and complex designs, identification of the appropriate level for tests and full reporting of outcomes
☐	☒	Estimates of effect sizes (e.g. Cohen's *d*, Pearson's *r*), indicating how they were calculated

Our web collection on statistics for biologists contains articles on many of the points above.

Software and code

Policy information about availability of computer code

Data collection	Participants were recruited using Prolific Academic, and took a survey administered in Qualtrics survey software.
Data analysis	All analyses were conducted in R (version 4.2.0).

For manuscripts utilizing custom algorithms or software that are central to the research but not yet described in published literature, software must be made available to editors and reviewers. We strongly encourage code deposition in a community repository (e.g. GitHub). See the Nature Portfolio guidelines for submitting code & software for further information.

Data

Policy information about availability of data

All manuscripts must include a data availability statement. This statement should provide the following information, where applicable:
- Accession codes, unique identifiers, or web links for publicly available datasets
- A description of any restrictions on data availability
- For clinical datasets or third party data, please ensure that the statement adheres to our policy

Data necessary to replicate these analyses is available at https://osf.io/ay9fc/.

Human research participants

Policy information about studies involving human research participants and Sex and Gender in Research.

Reporting on sex and gender	We designed our study in order to analyze differences in three dependent variables (discernment ability, metacognitive efficiency, and response bias) according to self-reported gender identity. In our final sample, there were 247 males, 252 females, and 1 individual choosing not to disclose their gender identity. All gender-based analyses are reported in the main text when performed. Our comparisons of interest were between males and females, and as such, the individual choosing not to disclose their gender identity was excluded from these analyses. We recruited 125 participants in four age bins (18-32, 33-47, 48-62, 63+), and each bin was balanced for political party (Democrat or Republican) and self-reported gender (male/female), resulting in approximately 30 participants per cell (i.e., ~30 Democrats who self-identified as male, ~30 Republicans who self-identified as female, etc.)
Population characteristics	See "behavioral and social sciences study design" section.
Recruitment	Participants were recruited via Prolific Academic. No obvious self-selection biases exist; participants may have elected to take our study instead of others due to personal interests and/or payment.
Ethics oversight	Northeastern University IRB.

Note that full information on the approval of the study protocol must also be provided in the manuscript.

Field-specific reporting

Please select the one below that is the best fit for your research. If you are not sure, read the appropriate sections before making your selection.

☐ Life sciences ☒ Behavioural & social sciences ☐ Ecological, evolutionary & environmental sciences

For a reference copy of the document with all sections, see nature.com/documents/nr-reporting-summary-flat.pdf

Behavioural & social sciences study design

All studies must disclose on these points even when the disclosure is negative.

Study description	This study examines individual differences in metacognitive ability when judging the veracity of news headlines that have circulated online in the past year. All participants will be presented with 70 true and 70 false headlines in random order and provide veracity and confidence judgments for each. Utilizing an approach grounded in signal detection theory, we aim to measure (1) how well individuals distinguish between true and false headlines, (2) whether or not they exhibit a general response bias in their veracity judgments, and (3) how metacognitively aware they are of their own discernment ability. We will also compare group differences in metacognitive ability across the demographic variables age, education, political party, and gender. Data are quantitative.
Research sample	Participants were recruited via Prolific Academic in four age groups (18-32, 33-47, 48-62, and 63+). We recruited 125 participants in each age bin, and each bin was balanced for political party (Democrat or Republican) and self-reported gender (male/female), resulting in approximately 30 participants per cell (i.e., ~30 Democrats who self-identified as male, ~30 Republicans who self-identified as female, etc.). As a result of this sampling strategy/study design, this sample was not representative.
Sampling strategy	Typical studies analyzing metacognitive differences involve psychophysical tasks, with small sample sizes and a larger number of trials. For instance, Rahnev et al. (2020) compiled 145 datasets investigating confidence and found that the median sample size was 37 participants with 309 trials. Given that exact effect sizes remain unknown, we based our sample size on similar studies. For instance, Scott et al. (2014) had 450 participants and 60-64 trials, and Sultan et al. (2022) used 760 participants for 37 trials (for news headlines). Thus, we decided that 500 people for 140 trials would be sufficient, giving us 125 participants in each of our age bins. This sample size is greater than other known studies comparing older and younger adults (for instance, N = 72, Dodson, Bawa, & Krueger, 2007 and N = 183 Prim & Moore).
Data collection	Participants from Prolific Academic viewed a survey constructed in Qualtrics. This study was administered online, and there were no experimentally manipulated groups.
Timing	Data were collected from August 15th to August 17th, 2022.
Data exclusions	Our a-priori exclusion criteria were participants who reported a lack of effort (N = 5) and did not answer all items (N = 15). We used the outlier labeling rule to exclude participants with extreme metacognitive efficiency and discernment values (N = 9)45. Finally, we removed participants with negative m-ratio values (N = 4)43. In our final sample (N = 500), there were 247 men, 252 women, and 1 individual choosing not to disclose their gender. Participants' age ranged between 18 and 84 (M = 47.2, SD = 16.4). Note that we excluded participants with negative or extreme m-ratio values when re-estimating metacognitive efficiency for our politically equated stimulus set (where we removed n = 9 additional participants; N = 491), and when calculating metacognitive efficiency for the political favorability analyses (where we removed n = 40 participants; N = 460).

Non-participation	Of the 551 participants who took the survey administered in Qualtrics, 9 did not complete it, resulting in a response rate of 98.3% (before exclusion criteria were applied).
Randomization	*If participants were not allocated into experimental groups, state so OR describe how participants were allocated to groups, and if allocation was not random, describe how covariates were controlled.*

Reporting for specific materials, systems and methods

We require information from authors about some types of materials, experimental systems and methods used in many studies. Here, indicate whether each material, system or method listed is relevant to your study. If you are not sure if a list item applies to your research, read the appropriate section before selecting a response.

Materials & experimental systems

n/a	Involved in the study	
☒	☐	Antibodies
☒	☐	Eukaryotic cell lines
☒	☐	Palaeontology and archaeology
☒	☐	Animals and other organisms
☒	☐	Clinical data
☒	☐	Dual use research of concern

Methods

n/a	Involved in the study	
☒	☐	ChIP-seq
☒	☐	Flow cytometry
☒	☐	MRI-based neuroimaging
